# Sources of psychological distress among primary care physicians during the COVID-19 pandemic’s first wave in Spain: a cross-sectional study

**DOI:** 10.1017/S1463423621000566

**Published:** 2021-10-18

**Authors:** Ana Cebrián-Cuenca, José Joaquín Mira, Elena Caride-Miana, Antonio Fernández-Jiménez, Domingo Orozco-Beltrán

**Affiliations:** 1 Centro de Salud Cartagena Casco, Cartagena, Murcia, Spain; 2 Health Psychology Department, Universidad Miguel Hernández, Alicante, Spain; 3 Centro Salud Almassera de Tonda, Vilajoyosa, Alicante, Spain; 4 Instituto de investigación del Hospital Clínico de Valencia (INCLIVA), Valencia, Spain; 5 Unidad Investigación, Dpto. San Juan de Alicante, Centro de Salud Cabo Huertas, Alicante, Spain

**Keywords:** COVID-19, infectious diseases, mental health, occupational/environmental medicine, occupational stress, stress

## Abstract

**Background:**

The COVID-19 pandemic is affecting people worldwide. In Spain, the first wave was especially severe.

**Objectives:**

This study aimed to identify sources and levels of distress among Spanish primary care physicians (PCPs) during the first wave of the pandemic (April 2020).

**Methods:**

A cross-sectional study was conducted using a survey that included sociodemographic data, a description of working conditions related to distress [such as gaps in training in protective measures, cleaning, and hygiene procedures in work setting, unavailability of personal protective equipments (PPEs) and COVID-19 RT-PCR test, and lack of staff due to be infected] and a validated scale, the ‘Self-applied Acute Stress Scale’ (EASE). The survey was answered by a non-probability sampling of PCPs working in family healthcare centres from different regions of Spain. Analysis of variance and multivariate linear regression analysis were performed.

**Results:**

In all, out of 518 PCP participants, 123 (23.7%) obtained high psychological distress scores. Only half of them had received information about the appropriate use of PPE. PCP characteristics associated with higher levels of distress include female gender [1.69; 95% confidence interval (CI) 0.54, 2.84]; lack of training in protective measures (1.96; 95% CI 0.94, 2.99); unavailable COVID-19 RT-PCR for health care workers after quarantine or COVID-19 treatment (−0.77 (−1.52, −0.02). Reinforcing disinfection of the work environment (*P* < 0.05), availability of PPEs (*P* < 0.05), and no healthcare professional was infected (*P* < 0.05) were related to the lowest distress score.

**Conclusions:**

A better understanding of the sources of distress among PCPs could prevent its effect on future outbreaks.

## Introduction

The COVID-19 pandemic is affecting people of all nations worldwide (Adams and Walls, 2020). In Spain, the epidemic has become especially severe, the reasons for which can be manifolded (Instituto de Salud Carlos III, 2020). By 23rd October, 2020, more than a million had been diagnosed and more than 50 000 people had died from COVID-19 (for a country of 46 million inhabitants).

Nevertheless, Spain had the highest life expectancy at birth among European nations (World Health Organization (WHO), 2017). In addition, the 2019 edition of the Bloomberg Healthiest Country index ranked it as the world’s healthiest country (Miller and Lu, 2019). It is debated that primary care in Spain is essentially provided by public providers, specialised family doctors, and stuff nurses, who provide preventive care to children, women, and elderly patients, along with acute and chronic care. However, Spain had 5106 confirmed cases/one million inhabitants, which is the highest rate all over the world (during the first outbreak, 29th, April 2020) (The Johns Hopkins University, 2020). As reported by the European Centre for Disease Prevention and Control, 20% of registered coronavirus cases in Spain were healthcare workers, compared with 10% in Italy, 3% in the USA, and 3.8% in China on those dates.

The pandemic has tested the Spanish health system and its professionals. Different stressors affecting primary care physicians (PCPs) such as burnout, working in crises, and other reported stressors have been reported. Shanafelt *et al.* (Shanafelt, Ripp and Trockel, 2020) have recently described eight sources of anxiety: lack of protective equipment, being exposed at work, not having rapid access to testing, an uncertainty that their organization will support care, access to childcare if schools are closed, support for other personal needs, provide competent medical care in different areas, and lack of information. The lack of protective measures, increased workload, and changing their roles rapidly may have caused them distress. Therefore, it is crucial to understand the specific sources of distress among PCPs in Spain to identify useful measures to reduce their psychological stress and to prevent it in future outbreaks. This study aimed to address the sources and level of distress among Spanish PCPs during the first wave of the COVID-19 outbreak.

## Methods

### Design, sampling, and recruitment

This was a cross-sectional online survey aimed to recruit a diverse non-probability sample of PCPs practicing in urban and rural settings across Spain. The survey was distributed in 2020 from 18 to 25 April via PCPs networks and two professional organisations.

Assuming a conservative estimation that 25% of the PCPs would rate a high score for psychological distress, the study would require a sample of at least 441 PCPs for estimating the expected proportion with 4% absolute precision and 95% confidence interval. Participation was voluntary and required informed consent.

In Spain (2019 data from the Ministry of Health), 29 086 family physicians work in primary care, 59% of women who work in some of the 13 133 health centres (mostly in rural areas), serving in a total population of about 46 million inhabitants. As the express method of accessing many PCPs and ensuring that the invitation to respond reached the entire country, we resorted to two organisations with PCP networks across Spain: the network for the study of diabetes in Primary Care (redGDPS) and the Spanish Family Physician Society (semFYC). Each organisation was requested to promote the survey at least once in their newsletter and/or website. The platform to respond was kept open until the required sample size was reached.

### Survey and data collection

We designed a survey titled ‘STREPRIC study’ STREss factors among PRImary Care physicians in Spain. Demographic characteristics, work conditions, and distress scale were investigated in this survey.

We explored some work conditions that could reduce distress such as if PCPs were trained to apply the adequate protective measures, their assessment about the cleaning and hygiene procedures, the availability of personal protective equipment (PPEs), and the availability of systematic reverse-transcription polymerase chain reaction (RT-PCR) testing (COVID-19 RT-PCR) for health care workers returning to work after quarantine or COVID-19 treatment, and if they or some colleagues were infected on those study dates.

The ‘Self-applied Acute Stress Scale’ (EASE) was used to determine the distress related to the care of COVID-19 patients among Spanish PCPs. The questions in this scale were asked about feelings, behaviours, and thoughts in the course of their professional work during the outbroke. The scores ranged from 0 to 30, establishing four ranges: 0–9 for good emotional adjustment, 10–14 for emotional distress, 15–24 for emotional overload, and 25 and above for extreme acute stress. This scale has been validated by our research group which considered the three phases to create a scale described by Boateng *et al.* (item development, scale development, and scale evaluation) (Boateng *et al.*, 2018). Its reliability, content validity, construct validity, criterion validity responsiveness, and interpretability were found to be appropriate (Mira *et al.*, 2021). Two-factor structure of the scale was confirmed for the core dimensions, affective responses, and fear and anxiety responses. Global scores of the norm group ranged from 3 to 30 (mean 10.0, SD 6.1, 95% CI 9.2–10.8). Average score in the affective responses was 6.0 (SD 3.9, 95% CI 5.5–6.5), and for fear and anxiety responses, it was 4.0 (SD 2.8, CI 95% 3.6–4.4).

The survey was administered using Google forms and was sent directly to PCPs from the two organisations mentioned (redGDPS and semFyC). Approval of the ethics was granted by the Ethics Committee of the San Juan University Hospital in Alicante.

### COVID-19 outbreak comparative data

As compared with other countries, on April 11, 2020, Spain had a mortality rate as high as 352/million inhabitants, Italy 329, France 201, The Netherlands 155, UK 147, Switzerland 118, USA 57, Iran 53, Portugal 46, Germany 33, Turkey 12, Brazil 5, and the world average rate was 14/million inhabitants^5^.

### Statistical analysis approach

Only cases with all items answered were considered. Categorical variables were analysed using the Chi-squared or Fisher’s Exact Test. ANOVA test was conducted to analyse the relationship between work conditions that could reduce distress and the EASE scores. A multivariate linear regression analysis was carried out, where the EASE score was considered as a dependent variable, and the factors included sex, age, setting (rural versus urban), and whether the PCPs had been trained to apply the adequate protective measures, and the availability of RT-PCR test. Data analysis was performed using SPSS v.26 statistical software.

## Results

A total of 518 PCPs responded to the survey. The majority were females (70.8) and those working in urban areas (71.4%). All the PCPs had observed their pattern of action change during the critical phase of the pandemic (moving to telephone care). During this period, care for patients with chronic conditions and home visits were reduced (Table 1).

**Table 1.** Descriptive analysis of the sample (April 2020, primary care physicians, *n* = 518)

Characteristics of primary care professionals		
Gender	*N*	%
Female	367	70.8
Male	151	29.2
Age		
<30 years	16	3.1
30–49 years	221	42.7
50–64 years	268	51.7
≥ 65 years	13	2.5
Health centre located in		
Rural	148	28.6
Urban	370	71.4
Training in protective measures		
No	281	54.2
Yes	237	45.8
**Changes in responsibilities during COVID-19 pandemic**		
I have done the same job	21	4.1
I have attended more emergencies	106	20.5
I have switched to phone support	518	100.0
I have done less home care	224	43.2
I have seen fewer patients with chronic diseases	374	72.2
**Type of personal protective equipment**		
Surgical mask	500	96.5
FFP1 mask	39	7.5
FFP2 mask	317	61.2
FFP3 mask	37	7.1
Face shields or goggles	337	65.1
Surgical gloves	449	86.7
Disposable gowns	374	72.2
Biocidal water-alcohol solution	494	95.4
**Who provided personal protective equipment**		
Health service	166	32.0
Health service and donations	300	57.9
Own contribution	45	8.7
Availability of systematic reverse-transcription polymerase chain reaction (RT-PCR) testing (COVID-19 RT-PCR) for health care workers returning to work after close contact quarantine or treatment following infection		
Yes	150	28.7
No systematically	372	71.3

### Training for using personal protective equipment

Approximately half of the PCPs were trained in the use of PPE (45.8%). Most of them received PPE thanks to the collaboration of entities that donated material (66.6%) (Table 1).

### Availability of RT-PCR testing

Only 150 PCPs (28.7%) stated that RT-PCR testing was routinely performed at their facility for professionals after quarantine or upon return to work after being affected by COVID-19.

### Distress experimented

The mean direct score on the distress scale was 10.31 points (SD 6.01, CI 95% 9.79–10.83) (Table 2). In all, 123 (23.7%) PCPs scored above 15 points. The main sources of distress included the fear of infecting the family upon returning home and not being able to disconnect from work after the workday was over (Table 2).

**Table 2.** Scores on the acute stress scale of primary care professionals during the first wave of the COVID-19 pandemic in Spain (April 2020, primary care physicians, *N* = 518)

	Mean (range: 0–3) ± SD	Proportion of responders answering levels 2-3
I don't know what to do or where to start	1.07 ± 0.84	27.8
I can't help but think of recent critical situations. I can't seem to disconnect from work	1.41 ± 0.91	44.7
I keep my distance, I resent dealing with people, I'm irascible even at home	0.96 ± 0.84	24.1
I feel that I am neglecting many people who need my help	1.16 ± 0.88	36.3
I have difficulty thinking and making decisions, I have many doubts, I have entered a kind of emotional blockage	0.82 ± 0.80	18.7
I feel intense physiological reactions (shocks, sweating, dizziness, shortness of breath, insomnia, etc.) related to thecurrentcrisis situation	0.91 ± 0.88	26.9
I feel on permanent alert. I believe that my reactions now put other patients, my colleagues or myself at risk	0.83 ± 0.89	23.1
The worry about not getting sick causes me a strain that is hard to bear	0.99 ± 0.92	27.8
I'm afraid I'm going to infect my family	1.70 ± 1.00	58.5
I have difficulty empathising with patients' suffering or connecting with their situation (emotional distancing, emotional anesthesia)	0.45 ± 0.68	8.9

Level 2: emotional overload.

Level 3: extreme acute stress.

Women and PCPs who had not received training in the correct use of PPEs or those who reported unavailability of RT-PCR testing experimented with the highest level of distress in the care of patients with COVID-19 (Table 3). PCPs working in rural areas reported the highest fear and anxiety regarding COVID-19 care. The improved cleanliness and hygiene of the health centre, availability of PPEs, and that no workers at the centre were infected helped to mitigate the distress. The origin of PPEs (the Health Service itself or the donations) did not affect the levels of distress.

**Table 3.** The association of higher level of distress and personal characteristics and training (linear regression analysis)

	Emotional response beta coefficient (95% CI)	Fears and anxiety response beta coefficient (95% CI)	Total score beta coefficient (95% CI)
Gender (female)	0.98 (0.30, 1.66)^a^	0.71 (0.14, 1.28)^a^	1.69 (0.54, 2.84)^a^
Age	0.18 (−0.44, 0.80)	0.25 (−0.26, 0.77)	−0.43 (−0.61, 1.48)
Working setting (rural)	0.36 (−0.34, 1.05)	0.48 (−0.10, 1.06)^a^	0.84 (−0.34, 2.01)
Lack of training in protective measures	1.13 (0.53, 1.74)^a^	0.83 (0.33, 1.34)^a^	1.96 (0.94, 2.99)^a^
Availability of COVID-19 RT-PCR for health care workers returning to work after close contact quarantine or treatment following infection	−0.35 (−0.79, 0.09)	−0.42 (−0.70, −0.53)^a^	−0.77 (−1.52, −0.02)^a^

a P-value <0.05.

PCPs who claimed to be the ones who undertook the task of cleaning their work environment were those who achieved the highest EASE score (*P* < 0.05), together with those who stated that they did not have adequate PPEs (*P* < 0.05) and those who were infected (*P* < 0.05) (Table 4).

**Table 4.** Measures that helped reduce distress responses (April 2020, primary care physicians *n* = 518)

	Emotional response (mean ± SD)	Fears and Anxiety Response (mean ± SD)	Total Score (mean ± SD)	*P*-value^*^
**Cleaning and hygiene**				
I clean my own work environment (*N* ** =** 42)	7.05 ± 3.39	5.81 ± 3.44	12.86 ± 6.26	*P* < 0.05
The usual cleaning measures are carried out (*N* = 98)	6.28 ± 3.79	5.01 ± 3.09	11.29 ± 6.50	
Recently cleaning has been reinforced and disinfection is done daily (*N* = 174)	6.30 ± 3.47	4.70 ± 2.98	10.99 ± 5.88	
Cleaning has been reinforced from the beginning of COVID-19 and disinfection is done daily (*N* = 204)	5.09 ± 3.39	3.65 ± 2.56	8.74 ± 5.46	
**Availability of PPEs**				
I do not yet have adequate measures (*N* = 26)	7.38 ± 2.62	5.81 ± 3.01	13.19 ± 4.99	*P* < 0.05
They have been coming in, but long after the start (*N* = 398)	5.82 ± 3.62	4.38 ± 2.96	10.20 ± 6.12	
I have it since the beginning of the crisis (*N* = 94)	5.71 ± 3.39	4.26 ± 2.86	9.97 ± 5.63	
**Leave of absence due to COVID-19 pandemic**				
Yes, I was on sick leave for COVID-19	6.90 ± 3.30	5.68 ± 3.28	12.58 ± 6.12	*P* < 0.05
No, but there have been doctors at my center who have been on leave for COVID-19	6.17 ± 3.62	4.65 ± 2.92	10.82 ± 6.03	
No, there have not been any doctors on leave from COVID-19 at my centre	5.00 ± 3.35	3.59 ± 2.69	8.59 ± 5.50	

SD = Standard deviation.

* Results of the ANOVA test used to gain information about the relationship between the EASE scores and potential sources of distress.

## Discussion

These results suggest that a quarter of the participants reported experiencing acute stress during the first wave, which was more intense when there was a perceived increased risk of SARS-CoV-2 infection. This indicates that many of them would find themselves in an emotionally distressing situation. Despite this emotional overload, most participants believed that they could continue to maintain their decision-making capacities as well as their abilities to empathise with patients.

During the COVID-19 pandemic, the usual dynamics of work in primary care (personalised and individualised attention in the clinic, follow-up by the same family doctor) were broken. Usual care was also discontinued, except in cases involving consultations, unproven emergencies, or common variable immunodeficiency-related pathologies (World Health Organization (WHO), 2020). The availability of PEP was reduced to decrease the risk of infection, especially in the early stages. All of them were distress precursors together with the fear of being infected or infected relatives.

### Personal protective equipment

Training and availability were crucial. Both failed according to the information provided by the participants- Less than half received specific training on the use and correct placement of PPEs. This contrasts with World Health Organization’s recommendations (World Health Organization (WHO), 2020) and the experience in previous outbreaks (Institute of Medicine, 2011; Nash, Jagger and Hogan Mitchell, 2014; Doll and Bearman, 2015; Tomas *et al.*, 2015; John *et al.*, 2016). These results have highlighted similar tendencies confirmed in previous studies on the emotional impact and the healthy work environment (Casas, Ramón Repullo and Lorenzo, 2002; Osca Segovia, Sanchez-Cabezudo and Garcia Castilla, 2006; Aguado Martín, Bátiz Cano and Quintana Pérez, 2013) and the training in the use of PPEs among healthcare workers should be one of the lessons learned to be prepared for future outbreaks. Participants in this study have confirmed that they had supplemented their PPEs through donations during the first wave of the COVID-19 pandemic. It should also be noted that on the date of the survey, i.e. 42 days after the state of emergency was declared, some health professionals still reported that they did not have adequate protective equipment. The lack of availability of adequate protection material, as well as the possibility that some of the donations received may not have passed through the adequate quality certification, could have contributed decisively to the high number of healthcare providers infected by COVID-19 in Spain, emphasising the fact that health professionals did not perceive increased stress because this material was donated.

### Distress experienced

Regarding the questions on the distress scale, it was highlighted that despite being capable of dealing with stressful situations, considering that such situations are part of their normal work routine, almost a quarter of the participants obtained a score higher, showing higher or extreme acute stress. These results underline the effect studied in other countries and samples of healthcare workers (Lai *et al.*, 2020; Pappa *et al.*, 2020; Qi *et al.*, 2020; Rossi *et al.*, 2020; Zhang *et al.*, 2020) also in primary care. Moreover, these results show a similar trend confirming the fear of infecting the family and not being able to disconnect at the end of the shift, the two consequences most often cited by professionals as signs of distress. The EASE scale has also been used in a study in four Latin American countries during their first wave. In that study an overall average score of 9.5, slightly lower than that found in this study (Mira *et al.*, 2020). The items score followed a similar profile confirming the most worries and sources of distress of healthcare workers during the first wave.

There are no previous studies comparing distress between workers in urban and rural settings. These results suggest higher responses of fear and anxiety among PCPs working in rural areas compared to those work in an urban setting.

### Mitigating distress

This study identified several factors that contributed to mitigating the level of perceived distress among the participants. This included availability of PPEs, reinforcement in the cleaning and hygiene tasks of the health care centres, and no workers at the centre were diagnosed COVID-19. Likewise, performing COVID-19 RT-PCR for PCPs returning to work after quarantine or COVID-19 treatment significantly reduced distress. These results confirm previous conclusions from studies conducted in other countries during the pandemic (Kisely *et al.*, 2020).

### Strengths

To our knowledge, this is the first study carried out to determine the emotional impact and perception of distress of PCPs during the management of the COVID-19 pandemic in Spain, as well as its relationship with the lack of adequate PPEs and specific protection measures required for this situation. This is a key aspect since it represents one of the groups with the highest number of people infected internationally by the COVID-19 pandemic. It is worth highlighting the large sample size, representing various PCPs with different conditions and workplaces involved in health care and distributed throughout the Spanish geography.

### Limitations

The objective of this study was not to achieve a representativeness of all the regions of the country, but to reach a minimum number of surveys that were diverse in a short time. Similarly, the survey was sent to PCPs linked to various scientific societies, who were especially motivated regarding the subject. There is a clear predominance of the female sex, a fact that may be due to the predominance of females among the healthcare professions and of those working in urban centres. The number of professionals who declined to respond could not be determined. It should be noted that there was no random sample of participants but a convenient one which limits the power of the study.

### Conclusions

A better understanding of the sources of distress among PCPs could prevent its effect on future outbreaks. PCPs need to feel protected that every effort is being made to achieve this target. The overload can be more difficult to manage when they do not feel needs are being met to deal with these outbreaks.

## Availability of data and materials

Data are not publicly available due to the lack of consent from participants to share it beyond this study.
